# Optical Tweezers Exploring Neuroscience

**DOI:** 10.3389/fbioe.2020.602797

**Published:** 2020-11-27

**Authors:** Isaac C. D. Lenton, Ethan K. Scott, Halina Rubinsztein-Dunlop, Itia A. Favre-Bulle

**Affiliations:** ^1^School of Mathematics and Physics, The University of Queensland, Brisbane, QLD, Australia; ^2^Queensland Brain Institute, The University of Queensland, Brisbane, QLD, Australia

**Keywords:** optical tweezers, light sculpting, neuroscience, neuronal dynamics, single molecules, brain connectivity, neurons, brain development

## Abstract

Over the past decade, optical tweezers (OT) have been increasingly used in neuroscience for studies of molecules and neuronal dynamics, as well as for the study of model organisms as a whole. Compared to other areas of biology, it has taken much longer for OT to become an established tool in neuroscience. This is, in part, due to the complexity of the brain and the inherent difficulties in trapping individual molecules or manipulating cells located deep within biological tissue. Recent advances in OT, as well as parallel developments in imaging and adaptive optics, have significantly extended the capabilities of OT. In this review, we describe how OT became an established tool in neuroscience and we elaborate on possible future directions for the field. Rather than covering all applications of OT to neurons or related proteins and molecules, we focus our discussions on studies that provide crucial information to neuroscience, such as neuron dynamics, growth, and communication, as these studies have revealed meaningful information and provide direction for the field into the future.

## 1. Introduction

Since the late 1980s, optical tweezers (OT) have been extensively used for studying biological cells and whole organisms (Ashkin and Dziedzic, 1987), the main reason being that OT allows the physical manipulation of biological structures and environments in a non-invasive way using only light. In addition, it is a highly flexible optical tool that can hold, displace, stretch, and spin a large variety of complex-shaped objects and assembles. However, OT has taken a long time to prove its usefulness in neuroscience, in part due to the complexity of the brain and the associated difficulties with trapping or imaging objects within it.

In recent years, there have been great advances in OT and its combination with other modern optical tools for manipulating complex objects, mechanically altering surfaces, and controlling dynamics. Consequently, OT has become a remarkable technique for studying the physical properties and intrinsic forces of neurons, their axonal navigation preferences and regeneration processes, as well as some of the fundamental dynamics around their function. As new technologies have emerged and been cleverly combined with OT, the precision and depth of OT manipulation has increased, opening new avenues for neuroscience studies. This has enabled studies into the core processes driving neuronal growth and function, and on the larger scale, the formation of networks and complex information processing. As such, OT has been, and continues to be, a valuable tool for exploring neuroscience.

In this review, we focus our attention on the application of OT in neuroscience: how OT answers fundamental questions in neuroscience, the important findings that OT has delivered to the field, and where and how OT can further drive neuroscience discoveries. In the next section (section 2), we provide a description of optical tweezers with a focus on their flexibility and large number of potential applications in physics and biophysics. Rather than provide a detailed introduction to optical tweezers, we direct our discussion toward aspects that make OT particularly useful for neuroscience. In section 3, we cover recent applications of optical tweezers in neuroscience that have already provided crucial information on neuronal dynamics, growth, and modes of communication. We are particularly mindful in selecting and discussing studies that do not simply apply OT to neurons (or their receptors and involved molecules), but provide new meaningful information and direction for neuroscience. In section 4, we discuss current trends in optical trapping for neuroscience and where the field is heading. In recent years, great effort has been directed toward improving the quality of optical traps, extending the size range of particles and molecules that can be optically confined, as well as toward achieving trapping and manipulation deeper within tissue and turbid media. Highlighting these advances, we discuss the new potential capabilities of OT and its future in exploring neuroscience.

## 2. Optical Tweezers

Optical tweezers (OT) can be used to apply precise and very localized optical forces to microscopic particles. Using only light, OT is able to influence the motion of objects in a non-contact way, as well as inside optically transparent cells or living organisms. Additionally, OT can be used to measure mechanical properties of cells and their environments: by either observing how a trapped particle behaves or observing the light scattered by the trapped particle it is possible to measure properties such as mechanical stiffness and viscoelacticity. As will be shown in following sections, this makes OT especially useful for studying neurons, as well as for holding and manipulating objects that are difficult to manipulate using more conventional means such as with mechanical tweezers or micro-pipettes. In this section, we provide an overview of OT. The aim of this overview is to introduce the basic concepts of optical tweezers and simultaneously show some of the different optical tweezers techniques that could be useful for experiments in neuroscience. For a more complete coverage of the topic, we would like to refer interested readers to relevant textbooks and recent reviews that more thoroughly cover the theory behind optical trapping (Ashkin, 2006; Jones et al., 2015; Pesce et al., 2015) and its applications in biological (Choudhary et al., 2019; Favre-Bulle et al., 2019) and non-biological (Muldoon et al., 2012; Li et al., 2019) contexts.

**Figure 1:**
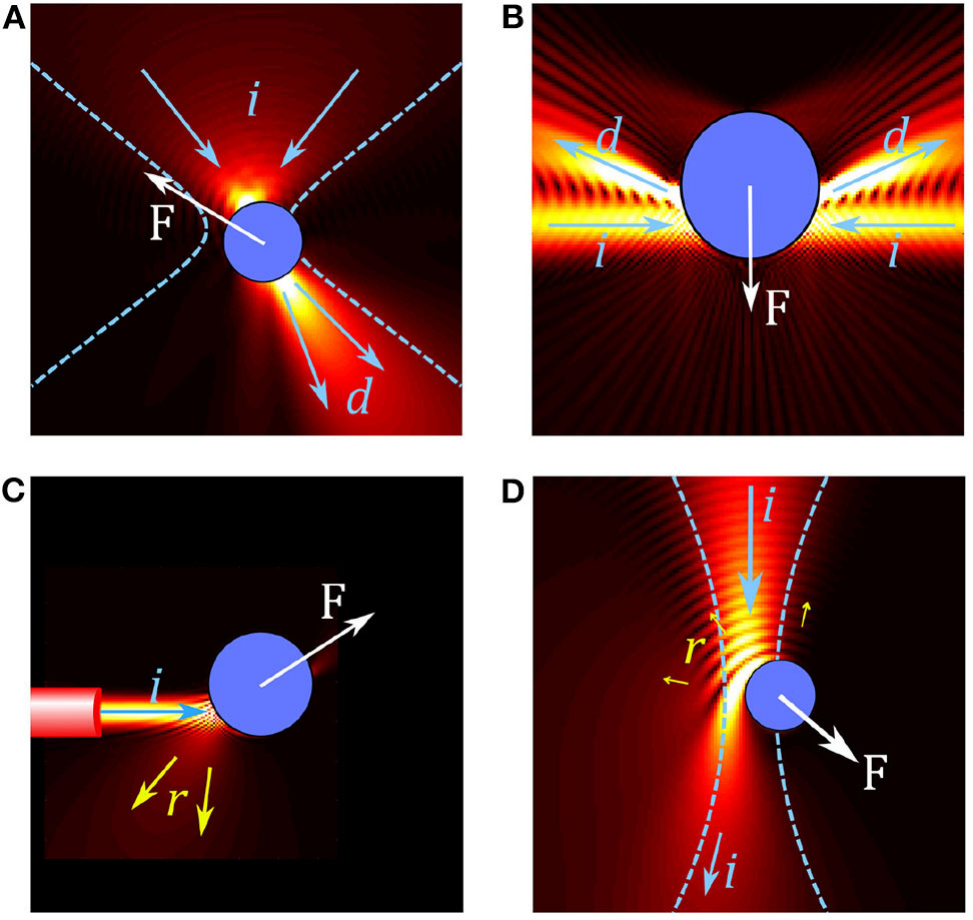
Overview of forces in different optical trap configurations. **(A)** In conventional single beam OT, a particle in a tightly focused Gaussian beam acts like a small lens, focusing and deflecting the beam. The resulting force *F* on the particle can be understood by considering the change in momentum between the incoming beam (illustrated by arrows *i*) and the deflected beam (arrows *d*). **(B)** Two weakly focused counter propagating beams are deflected by a particle, resulting in a gradient force on the particle. **(C)** A diverging beam from the end of an optical fiber reflects (arrows *r*) from a reflective particle, resulting in a scattering force which pushes the particle. **(D)** Simulation of an absorbing particle in a Gaussian beam; some light is reflected but most of the light is absorbed, leading to a large absorption force.

Optical tweezers use light to trap and manipulate small particles. The most common OT configuration involves using a highly focused laser beam, usually in the visible to near-infrared wavelength range (i.e., between 0.5 and 1 μm). At the beam focus, small particles can become trapped when the optical forces are large enough to overcome the other forces acting on them such as Brownian motion or fluid drag. The main optical forces in OT are: **the scattering force**, which arises from light reflecting off the particle and acts to push the particle in the direction of the beam propagation; **the gradient force**, which is related to the change in optical field intensity and acts to pull (or push) the particle toward (or away from) most intense regions of the laser light (depending on the particle's optical properties); and **the absorption force**, which arises from light being absorbed by the particle and typically behaves similarly to the scattering force but can also lead to other interesting thermal effects. As illustrated by the examples of optical trapping configurations shown in Figure 1, these forces, their magnitude, and the dominant forces depend on the properties of the OT system (including wavelength, coherence, and beam shape) as well as the properties of the particle (refractive index, size, absorption).

**Figure 2:**
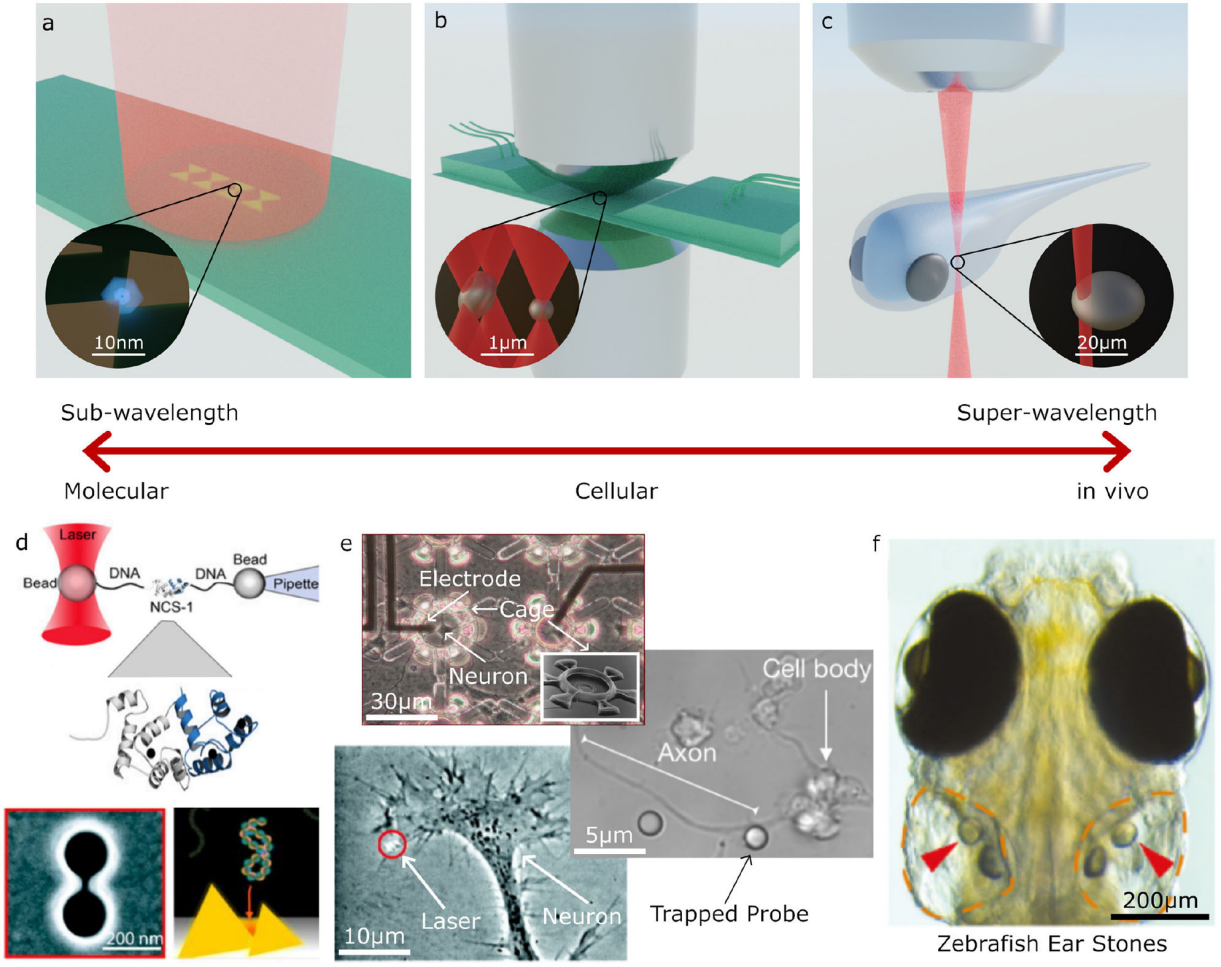
Examples applications of Optical Tweezers. **(a–c)** Different optical trapping experiments for trapping sub-wavelength to super-wavelength sized objects: **(a)** plasmonic bow-tie antenna (gold) illuminated by a weakly focused beam holding a sub-wavelength sized particle, **(b)** inverted microscope and microfluidic chamber used for holographic (multi-beam) optical tweezers with a high numerical aperture condenser to collect the scattered light for direct optical force measurement, **(c)** optical trapping an otolith (ear stone) inside a zebrafish. **(d–f)** Examples of molecular, cellular and large scale trapped objects and applications: **(d)** molecular sized particles can be manipulated with auxiliary particles or specially designed plasmonic structures; **(e)** on a cellular scale, OT can be used to place cells inside structures, manipulate parts of cells, or for indirect manipulation with probe particles; **(f)** at larger scales, OT can be used for manipulating structures inside living organisms, such as an otolith inside a zebrafish **(d–f)** adapted/reproduced from: Favre-Bulle et al. (2019) (CC BY 4.0); Rodríguez (2019) (CC BY-NC-ND 3.0 CL); Ehrlicher et al. (2002) Copyright 2002 National Academy of Sciences; Heidarsson et al. (2014) Copyright 2014 the authors; Pine and Chow (2009) Copyright 2008 IEEE, reprinted with permission; Pang and Gordon (2012) and Shoji et al. (2013) Copyright 2012, 2013 American Chemical Society; scale bars have been added to show approximate scale).

Optical trapping can be broadly split into three regimes for trapped particles of sub-wavelength, wavelength, and super-wavelength sizes; these approximately correspond to manipulation at the molecular, cellular and whole organism scales, respectively. The types of optical tweezers systems used in these three regimes also varies greatly. Figure 2 shows several examples of biological systems in these different size regimes which can be studied with optical tweezers and examples of the optical tweezers systems often used for these studies. For trapping sub-wavelength sized particles it is often necessary to use auxiliary particles or plasmonic structures to enhance the forces acting on the particle, as depicted in Figures 2a,d. The most commonly used designs closely resemble a regular optical microscopy system with an objective, condenser, camera, and illumination for imaging as well as a tightly focused laser beam for optical trapping. Two examples of these systems are depicted in Figures 2b,c. In these single beam systems, the dominant force is typically the gradient force or, if the particle is very reflective, the scattering force. Three dimensional trapping is achieved only when the gradient force overcomes the scattering and absorption forces. This is usually achieved by using a highly focused beam in order to create a large intensity gradient around the beam focus and often results in systems with a very small working distance (Figure 2b). However, it is also possible to use lower numerical apertures and objective with much longer working distances (Figure 2b), either by only trapping in two dimensions or using counter propagating beams, as depicted in Figure 1B. With these systems, it is possible to manipulate particles in the 100 nm to 10 μm range with forces of the order of piconewtons (pN, 10^−12^ N) as long as the particles are not too reflective or absorptive. High absorption is not desirable in biological systems as it leads to substantial heating and subsequent destruction of the system under study.

Trapping of reflective or absorbing particles tends to be more difficult: the scattering and absorption forces tend to dominate over the gradient force, pushing particles along the beam propagation direction and out of the trap, as illustrated in Figures 1C,D. For reflective particles it is common to use counter-propagating traps (Figure 1B) or traps with multiple beams coming from different directions to reduce the effect of the scattering force (Zhao, 2017). Another approach is to use structured light fields to reduce reflection in order to increase the depth of the optical trap (Taylor et al., 2015). While absorption related forces are regularly used for trapping non-biological particles, such as for driving micro-machines (Villangca et al., 2016) or for photophoretic trapping in air (Gong et al., 2016), most biological studies involving OT avoid using absorption related forces for direct manipulation of samples as the energy absorbed can lead to unwanted thermal effects or damage to the sample as mentioned above.

The most common way to reduce absorption is to choose a laser wavelength in the near infrared (IR) region (commonly used wavelengths include 980 and 1,064 nm) (Palima et al., 2015). Svoboda and Block (1994) provide a discussion of general considerations for studies involving biological specimens: the authors suggest choosing wavelengths at the near-IR end of the spectrum between 750 and 1,250 nm. IR and near IR wavelengths (above 750 nm) tend to be a good choice for OT in cell biology since they minimize light absorption by organic molecules such as: proteins, nucleic acids, carbohydrates, and lipids. However, above 1,250 nm light tends to be strongly absorbed by water. Hence, near IR (750–1,250 nm) tends to be a good tradeoff for OT with biological specimens; and from a historical point of view, 980 and 1,064 nm wavelengths have been popular due to their relatively low cost and good performance. Due to the complexity of cells and biological materials, and the various criteria for cell damage, it is difficult to provide general statements about absorption or recommendations for laser wavelength and intensity. When considering a particular cell, it may be important to consider the different materials in the cell, how they absorb light, and the appropriate criteria for cell damage within the context of neuroscience. Accurate determination of any effects of light radiation is crucial in all experiments involving biological matter. Control experiments are usually performed in addition to the experiments involving OT, in order to discriminate between the effect of the light from the probe manipulated by light. When the use of control measurements is not appropriate, simply trying OT with a particular sample and looking for obvious indications of heating (such as increased thermal motion, cavitation, or burning) can still provide useful information on the limits in wavelength and intensity that the system can handle. Choosing an appropriate wavelength that is not absorbed should be the first priority for avoiding absorption related cell damage and heating; however, as with reflective particles, using structured light fields or multiple beams can be useful for achieving large optical forces with lower beam powers, reducing the likelihood of cell damage. By structuring the illumination, light can be distributed evenly throughout a sample or structured to avoid certain regions of a sample (Zhang and Milstein, 2019; Zhang et al., 2019).

**Figure 3:**
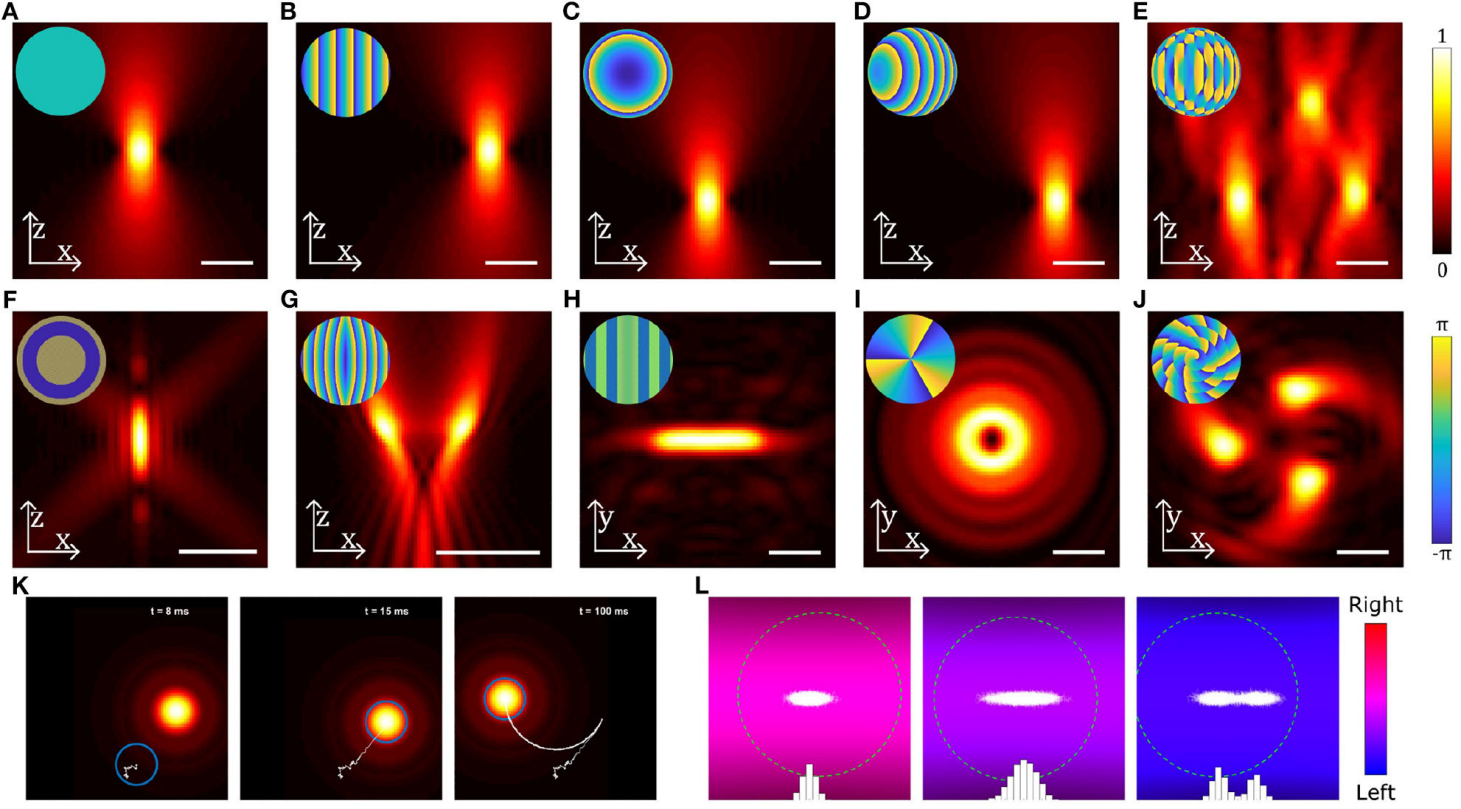
Examples of different beams used in optical trapping. **(A–J)** Near-field intensity patterns and corresponding far-field phase patterns (insets) for various static beams: **(A)** Gaussian beam, **(B)** a linear grating used to shift the beam in the radial direction, **(C)** a Fresnel lens used to shift the beam in the axial direction, **(D)** combination of b and c to shift the beam in an arbitrary direction, **(E)** multiple OT created by combining gratings for each beam using the Prisms and Lenses algorithm (Reicherter et al., 1999), **(F)** Annular beam, **(G)** Tug-of-war beam (Bezryadina et al., 2016), **(H)** Line shaped trap, **(I)** Beam carrying orbital angular momentum, **(J)** Chiral beam using annular subzone vortex phase plate (Yang et al., 2018). **(K)** Still images from Supplementary Video 1 showing a particle (blue circle) initially outside a moving OT, falling into the OT and then being dragged by the OT. Particle displacement track is shown in white. **(L)** Still images from Supplementary Video 2 showing the distribution of positions (white dots and blue bars) of a particle (green dashed circle) in a counter-propagating OT using feedback in the horizontal direction: the three panels show (from left to right) a trap with a high stiffness, a trap with a low stiffness and a trap with two equilibria, all generated using the feedback system. Scale bars show 2 μm; particles in **(K,L)** are 0.4 μm radius spheres. Beams have been generated and simulated using OTSLM (Lenton I. C. D. et al., 2020b) and the Optical Tweezers Toolbox (Lenton, 2020a), see supplemental code for more information.

Manipulating biological entities is often not possible with single Gaussian beam optical tweezers. With structured light, it is possible to create multiple beams or beams with different shapes which enable optimal light matter interaction with biological systems. Structured light fields can be created by modifying the phase or intensity of the trapping beam or both. In single beam OT, this is typically done by using optical elements such as lenses, mirrors, or phase masks placed before the focusing objective. One of the most configurable methods for modifying the phase/intensity of the incident beam is a computer controlled spatial light modulator (SLM) (Curtis et al., 2002). Using an SLM, the phase and/or intensity of the incident beam can be rapidly modified to create multiple traps or structured optical fields for trapping and orientating particles (Bowman et al., 2014; Lenton I. C. D. et al., 2020b). Figures 3B–J shows examples of different beams that can be generated by modulating the incident beam shown in Figure 3A, either by using an SLM or suitable combinations of mirrors/lenses/masks. Using a combination of relatively simple patterns (Figures 3B–D) a single beam can be split into multiple traps (Figure 3E) that can be controlled independently or used together to manipulate different parts of a large particle or organism. The number of traps is primarily limited by the available laser power and damage threshold for the beam shaping components, but with modern lasers it is possible to achieve 10 to 100 s of traps with either static or time-averaged configurations. Beams carrying orbital angular momentum (Figure 3I) or spin angular momentum can be used to rotate particles (Simpson et al., 1997; Grier, 2003; Favre-Bulle et al., 2019), and structured light fields can be used to orient or stretch particles (Figures 3F–J) (Bezryadina et al., 2016; Lenton I. C. D. et al., 2020a). If the devices used to generate these patterns are fast enough, beams can be dynamically scanned to create time averaged potentials or move particles around (Supplementary Video 1, Figure 3K). By tracking the particle position, OT can be implemented with feedback systems that can be used to stabilize its motion within traps or create traps with adjustable trap stiffness or multiple equilibria, as shown in Supplementary Video 2 (Figure 3L).

Conventional OT requires focusing a laser beam down to a tightly focused spot using a microscope objective. When the particle is deep within a scattering medium, such as skin or brain tissue, this can make focusing more difficult and limit the application of conventional OT. One solution is to use adaptive optics and other advances from imaging in order to be able to focus light deep within a sample (Wang et al., 2015; Hofmeister et al., 2020). Another alternative is to use non-conventional OT, such as OT created at the end of optical fibers (fiber optical tweezers, FOT) (Constable et al., 1993; Liu and Yu, 2017), as illustrated in Figure 1C. While traditional OT systems are bulky, FOTs offer the advantage of being miniaturized, self-sustaining system, with optical traps created in 3D and calibrated *in situ*. Using a regular single-mode optical fiber, FOT can be used much like conventional single beam OT except the generated beams are often not as tightly focused, and, as a consequence, the gradient forces are often much weaker. In order to achieve stable trapping, FOT (Chiang et al., 2019) is often used in a counter-propagating configuration (Bellini et al., 2010; Kreysing et al., 2014). A combination of structured light fields and fibers, including tapered fibers (Liu et al., 2006), fibers with coated or etched tips (Rodrigues Ribeiro et al., 2017), and multi-mode fibers combined with SLMs (Leite et al., 2017) can potentially lead to better trapping deep within scattering media.

All the techniques described so far focus on manipulating particles in the wavelength to super-wavelength range (≳100 nm) which can often be manipulated directly using tightly focused beams. The diffraction limit puts a restriction on the minimum spot size achievable in conventional OT, this makes manipulating sub-wavelength sized particles (~1–100 nm) more difficult. One solution is to use larger auxillary particles as handles (Heidarsson et al., 2014; Soltani et al., 2014), for example, Figure 2d illustrates how a single molecule can be manipulated using OT by tethering the particle to two larger probe particles using strands of DNA. Another approach is to use the fields generated near the surface of plasmonic antennas or at the surface of waveguides (Choudhary et al., 2019), as illustrated in Figures 2a,d. Unlike conventional OT, the fields generated by these structures can be highly localized with features smaller than the diffraction limit in the surrounding medium, allowing the trapping and study of individual molecules.

OT are capable of applying very precise piconewton scale forces to small particles, which makes them extremely useful for manipulation, such as for fast and precise placement of cells inside plastic microstructures (Pine and Chow, 2009). Being able to apply precise optical forces to particles also makes OT a useful tool for precise measurement of forces: by applying a known optical force to a particle, we can infer the non-optical forces acting on the particle based on its behavior. This idea is similar to atomic force microscopy (Neuman and Nagy, 2008) and is referred to as optical force microscopy or photonic force microscopy. Applications of optical force microscopy include studies of object profiles down to low nm resolution (Friese et al., 1999; Volpe et al., 2007; Pollard et al., 2010). Another example of the usefulness of precise force measurement is for studies of biological swimmers; for example, if we hold a swimming cell in an optical trap and gradually lower the trap power until the cell escapes, we can infer information about the swimming force from the optical force at the time the particle escaped (Nascimento et al., 2008). The range of forces OT can be used to measure is related to the range of forces that the OT can apply, i.e., OT can be used to measure piconewton and femtonewton scale forces, such as those encountered protein folding (Bustamante et al., 2020) or cell motility (Arbore et al., 2019; Armstrong et al., 2020).

Methods for calculating optical forces can be approximately grouped into two categories: inference based methods, which often involve calculating the force as a function of position in the trap using a particle of a known size; and direct force measurement methods, which involve estimating the optical force directly from the scattered light distribution (Fällman et al., 2004; Farré and Montes-Usategui, 2010; Jun et al., 2014; Thalhammer et al., 2015; Català et al., 2017; Bui et al., 2018). While both methods need to be calibrated, when and how these difference methods are calibrated can vary significantly. Unlike position based force measurement, direct force measurement requires collecting a significant amount of the total light scattered by a trapped particle. When the particle is weakly scattering, most of the light is forward scattered and the light can be captured using a high numerical aperture condenser (set-ups typically look similar to Figure 2b with a very short working distance). Direct force measurement with more strongly scattering particles requires more sophisticated optical set-ups. In comparison, position or calibration based force detection systems are relatively straightforward, requiring only a camera to track the particle's position. However, OTs are not always linear, and position/interference based methods often need to be calibrated for each individual particle in order to account for variations between samples. Being able to measure forces and hold particles makes OT an extremely useful tool for studying the environment surrounding particles, for example, for measuring temperature (Kashchuk et al., 2017) or viscoelasticity (Brau et al., 2007; Gibson et al., 2017; Robertson-Anderson, 2018).

Conventional OT systems have become relatively routine to set up (Pesce et al., 2015), and in many cases they can be integrated into existing microscope systems (Candia et al., 2013) or bought as complete kits from various optics manufacturers (2020c; 2020d). As it is an optical technique, it is relatively easy to combine OT with existing microscope systems or other spectroscopy and imaging technologies such as fluorescence, Raman, or phase contrast (Gong et al., 2018; Kashekodi et al., 2018). For example, OT have been demonstrated to be compatible with different electrophysiology and electrode array systems which can be useful for stimulating or monitoring neurons (an example of which is shown in see Figure 2e) (Pine and Chow, 2009; Difato et al., 2011). On the other hand, due to their small size the developments in plasmonic and waveguide based tweezers (Soltani et al., 2014; Choudhary et al., 2019) offer the potential for integration with *lab on a chip* systems or for use *in vivo*. In the cases where the forces created by OT aren't enough to completely confine a specimen, OT have been combined with other trapping technologies such as acoustics, magnetic tweezers, microfluidics, and mechanical systems (Wuite et al., 2000; Neuman and Nagy, 2008; Thalhammer et al., 2016; Dholakia et al., 2020). OT share a lot of similarities with other systems which use tightly focused beams, including laser scissors (Greulich, 2007, 2017; Difato et al., 2011; Berns, 2020) and two photon photopolymerization systems (Grier, 2003; Chizari et al., 2019). By using either different wavelengths, pulsed beams, or simply turning up the laser power, OT systems can be adapted for cutting cells, performing microsurgery (Berns, 2020), and fabricating microstructures for use as probes or OT operated micro-machines (Chizari et al., 2019).

## 3. OT in Neuroscience

As discussed in the previous section, OT has proved to be an efficient tool for the optical manipulation and probing of transparent objects on the micro- and nanometer scale. These capabilities are particularly profitable for research in biology, where minimal disturbance of biological systems is required and the visualization and quantification of properties and dynamics is highly valuable. Consequently, shortly after the introduction of OT in biology by Ashkin and Dziedzic (1987), the first manipulations of cell organelles and chromosomes were performed (Berns et al., 1989), and quickly, OT became widely used in biology. OT can now be efficiently applied to cells (Zhang and Liu, 2008), organelles (Morshed et al., 2020), and molecules studies (Svoboda et al., 1993; Fazal and Block, 2011; Ritchie and Woodside, 2015). Comprehensive reviews on the application and evolution of OT in biology can be found in the literature (Molloy and Padgett, 2002; Berns and Greulich, 2007; Ashok and Dholakia, 2012; Difato et al., 2013; Favre-Bulle et al., 2019). In this section, we will focus our discussion on how OT can be applied to neurons (Kandel et al., 2000) and what valuable information has OT brought to neuroscience research.

**Figure 4:**
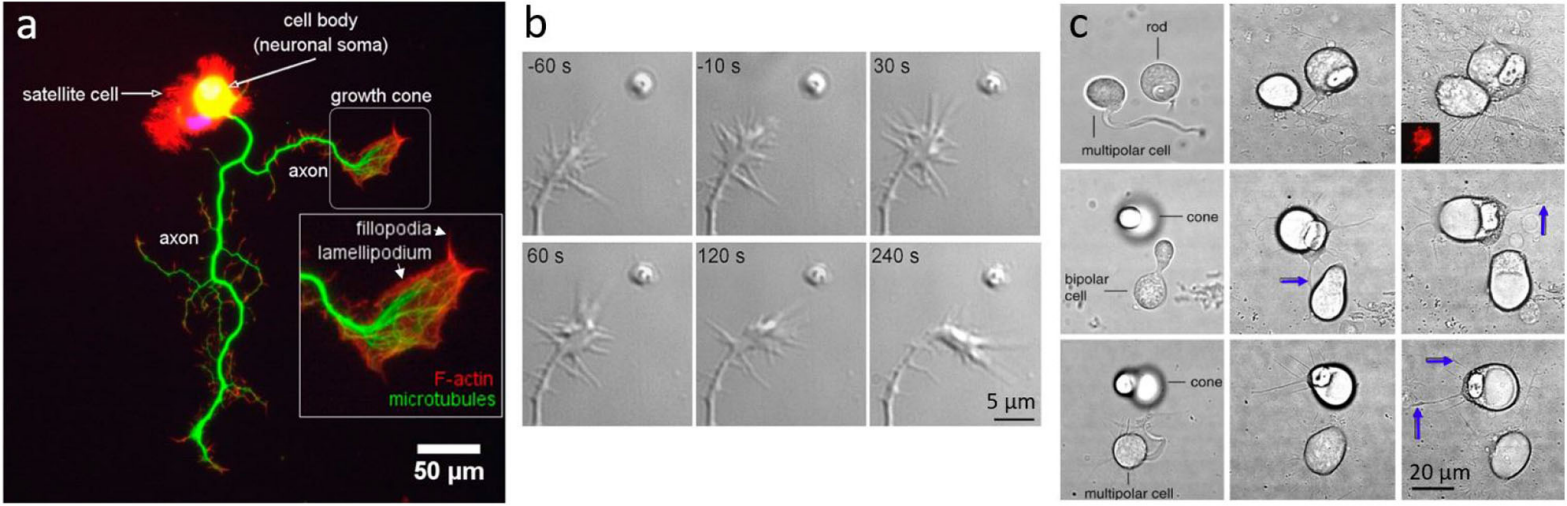
Neuronal growth and dynamics. **(a)** Fluorescent imaging of a neuron highlighting the macrostructure of the growth cone. Adapted from Muñoz-Lasso et al. (2020) (CC BY 4.0). **(b)** Neurite guidance by Sema3A released from a micrometer sized liposome from Pinato et al. (2012) (CC BY-NC-SA 3.0) **(c)** Axonal growth between pairs of cone, rod or bipolar cells, placed in proximity with OT: Left column shows cells immediately after OT manipulation, middle column shows interactions and axonal growth (blue arrows) after 3 days *in vitro*, and right column after 7 days *in vitro*; from Clarke et al. (2008) (CC BY-NC-ND 3.0).

Neurons have soma ranging typically between 10 and 30 μm in size, synapse buttons of few microns in diameter, and membrane receptors around 5 nm in size (Figure 4a). At these size scales, OT is an ideal tool both for direct manipulation of whole neurons (Townes-Anderson et al., 1998; Pine and Chow, 2009) or for indirectly probing synapses and receptors using auxiliary particles (Rodríguez, 2019) or plasmonics (Miyauchi et al., 2016).

### 3.1. On the Molecular Scale

A simplistic model of neuronal functioning would state that neurons compute information through the transport of neurotransmitters and ions flow. However, the large variety of molecules that influence the dynamics of neurons, or the processing of neural information, is overwhelming. On the molecular scale, these complex interactions remain largely mysterious, and OT has proved to be an excellent tool to probe structural dynamics and reveal some of the fundamental interactions for a large variety of molecules, including chains of nucleic acids, neural secretory molecules, receptors, and membrane proteins (Wang et al., 1997; Zahn and Seeger, 1998; Winckler et al., 1999; Imanishi et al., 2006; Neuman and Nagy, 2008; D'Este et al., 2011; Choudhary et al., 2018; Sonar et al., 2020). The key advantages of using OT for the study of molecules is its remarkable time and spatial resolution. High time resolution is particularly essential in single molecule force spectroscopy and allows to solve molecule kinetics and molecule binding otherwise impossible to study.

Molecular dynamics, such as folding or binding processes, depend on the structural arrangement and symmetrical disposition of atoms, or, on the larger scale, of sub-parts of the molecule. Advanced developments in OT have shown that atomic-scale resolution can be achieved, allowing the precise determination of structural changes (Kellermayer et al., 1997; Zhang et al., 2013; Bustamante et al., 2020; Sonar et al., 2020). A common method to observe such dynamics is to attach the molecule of interest to an optically trapped bead (or beads) and apply variable tension to the molecule by varying the bead position. The bead acts as a “handle” for exerting optically controlled forces. The other end of the molecule is either attached to a fixed substrate or a second bead held in a separate optical trap. As the molecule unfolds or changes its structural state under the tension applied on its end, the force curve of the trapped bead abruptly changes in a typical saw-tooth manner. The quantification of these force measurements can provide information such as the number of amino acid or nucleotides involved, the number of states, as well as the full energy profile of the molecule structural arrangements, which includes the free energy of each state and the energy barriers between states. One remarkable example of using this technique is the study by Brower-Toland et al. (2002), where they measured the successive release of individual nucleosomes in folded DNA. Interestingly, the analysis of the force detection revealed that a nucleosome is released in three steps, each step involving a partial unwrapping of the DNA. An example of using OT to reveal molecular dynamics for neuroscience is the study of the folding mechanisms of NCS-1, an important protein for neurotransmitter release. In a study by Heidarsson et al. (2013), they used OT and molecular dynamics to study the precise folding mechanism of the human NCS-1. The results revealed two intermediate folding structures of NCS-1 induced by calcium binding, and an interdomain folding dependence, presenting NCS-1 as a complex folding mechanism, compared to structurally related proteins.

A commonly used method based on OT to study the role and effects of different types of molecules on neurons is the manipulation of coated particles with the molecule of interest and its positioning to precise locations on neurons (Giannone et al., 2003; D'Este et al., 2011). An interesting example is the study of the regulation of secretory molecules in neurons. D'Este et al. (2011) used OT to hold micron sized particles coated with brain-derived neurotrophic factor (BDNF): a neurotransmitter modulator involved in neuronal plasticity and a mediator of activity-dependent dendrite branching. They have been able to coat particles with the secretory molecule and place them at specific sites on the dendrites of cultured hippocampal neurons of rat. The results show a significant increase of induced calcium signaling in the stimulated dendrite over on a long time period (up to 40 min), as well as an influence on the development of neurons. These results present OT as an appropriate method for a long-term and localized stimulation of specific sub-cellular neuronal compartments.

Another interesting study is the investigation of diffusion barriers in the plasma membrane. Nakada et al. (2003) used OT to directly drag single molecules and verify the presence of diffusion barriers in the axonal initial segment membrane from newborn rats. The results proved that a diffusion barrier does exist, and that this barrier is formed in neurons 7–10 days after birth.

### 3.2. Communication Modalities of Neurons

Another extremely active area of biology is the study of information transport and communication modalities between cells. Similarly, in neuroscience, great efforts are put into revealing the precise temporal and spatial dynamics of communication pathways in neurons. Neurons have been found to receive and transmit information through mechanical (mechanotransduction), electrical (action potential), and chemical (neurotransmitters) signals.

In a neuron-neuron communication scenario, the action potential within a neuron triggers the release of neurotransmitters to the next neuron, which changes the membrane potential of the receiving dendrites, building up toward either creating or suppressing an action potential within the receiving neuron. OT has been able to provide significant information to the investigation of neurotransmitter transport dynamics.

Studies in this area have focussed on the main actor in neurotransmitter release: synaptic vesicles (van Niel et al., 2018). These spherical membrane structures encapsulate neurotransmitters within axon terminals and fuse with the presynaptic membrane to release neurotransmitter into the synaptic cleft, thus influencing the physiology of the postsynaptic dendrite. The temporal and spatial dynamics of vesicle-cell interactions remains unclear, however, in a recent study by Prada et al. (2018b), they used OT to directly manipulate single extracellular vesicles produced by glia cells to study glia-to-neuron interaction. In particular, they looked at the transfer of miR-146a-5p: a protein involved in inflammation and immune function which play a significant role in dendritic spine formation and synaptic stability. Using OT, they moved the vesicles onto neurons and studied the effect of miR-146a-5p on dendrites and synapse population. They showed evidence that prolonged exposure to the inflammatory vesicles leads to a significant decrease in dendritic spine density which is also accompanied by a decrease in the density and strength of excitatory synapses. Prada et al. (2018a) later showed, using the same method, the first direct evidence of glia-derived vesicles fusion with the neuron plasma membrane, and that this fusion also occurs along neuronal processes. These important findings help elucidate the complex pathways of communication that are mediated by vesicles. However, further studies on *in vivo* models are necessary as they would allow the tracking of vesicles at the different stages of the process: through their biogenesis, transit routes and, finally, their delivery.

Neurons can also respond to mechanical stimuli by converting them into biochemical signals in a process known as mechanotransduction. This process is of fundamental importance for cells as they need to constantly adapt to the continuous reorganization and mechanical stress from the extra-cellular matrix and microenvironment. The importance of mechanical cues in controlling cell function has been acknowledged only recently (Handorf et al., 2015), and significant studies in the area still need to be undertaken in order to reveal the interactions among different mechanobiology pathways, which at the moment appear as complex entangled processes (Martino et al., 2018).

Since OT can apply forces and mechanical stimuli on the micro scale, it can be used to study mechanotransduction in cells (Wang et al., 2005) including neurons. Falleroni et al. (2018) have optically manipulated particles in oscillatory optical trap and applied piconewton forces perpendicularly to the cell membrane of mouse neuroblastoma NG108-15. Using this method they produced oscillatory membrane indentations and induced biochemical responses in the mouse nerve cells. They showed that very low levels of mechanical stress (5–20 pN) are sufficient to induce biochemical responses such as cellular calcium transients, and that the stimulus strength and the number of pulses affected the responses.

Using the same method, Bocchero et al. (2020) applied piconewton forces to rod cells in frogs. Interestingly, they showed that rods express channels that can be activated by direct mechanical stimulation, and are therefore mechanosensitive. Past studies have shown that rods, under strong illumination, expand, or shrink in length by few micrometers (Hardie and Franze, 2012; Lu et al., 2018), which indicates the existence of mechanical machinery within rod cells. Using OT, Bocchero et al. (2020) have therefore confirmed this hypothesis.

We further discuss the method of indentation with OT in section 3.4 on mechanical properties of neurons. We also discuss potential studies on mechanotransduction network in the outlook (section 5).

### 3.3. Growth and Dynamics of Neurons

Developmental neuroscientists have spent decades describing how neurons attain their mature architectures and identify their synaptic partners. Neurons have been shown to grow, extend their axons over great lengths, and wire up to neighboring neurons and sensory organs in order to create an extremely intricate computational network.

One fundamental question is how growing axons steer toward their targets. In order to study such complex processes, experiments are typically performed *in-vitro* and on very young neurons (1–7 DIV), when neurons are in the developing stage. One method recently used is the delivery of molecules encapsulated in liposomes, manipulated with OT, and directly delivered to neurons (Leung and Romanowski, 2012; Amin et al., 2016; Nguyen et al., 2019). This method allows precise delivery (micro to nano scale) with an amazingly precise number of molecules released locally. Pinato et al. (2012) used this method to study the effects of axon guidance molecules, such as Sema3A and Netrin-1, on the dynamics of the growth cone, a highly motile structure that controls the steering of the growing axon. They found that <5 Netrin-1 molecules initiate growth attraction, while 200 Sema3A molecules are necessary for growth repulsion (Figure 4b). This method allowed a highly precise delivery of molecules in space and in time, and can be used for the study of the effects and interaction of any molecules with neurons, with the exception of membrane-permeable molecules.

OT has also been used to investigate the mechanical properties of neurons' membranes. While neurons are not expected to be mechanically active, as muscles or fibroblast cells are, they have been found to be surprisingly osmotically and mechanically resilient, undergoing dramatic shape and volume changes (Bray et al., 1991; Wan et al., 1995). A tension hypothesis for surface area regulation in cells is “When membrane tension goes high locally, [surface area] is added locally from widespread, mechanically accessible endomembrane reserves. When tension goes low locally, excess [surface area] is retrieved locally” (Morris and Homann, 2001). While some studies have relied on surface deformation to support this hypothesis (Waugh et al., 1992; Evans and Yeung, 1994), Dai et al. (1998) have used OT to directly measure the tether force on a particle attached to the membrane surface mollusc neurons. As neurons were shrinking and swelling over time, the changes in forces were measured with OT and the membrane tension was calculated. The results suggest that the variations in membrane bending stiffness during cell swelling and shrinking was constant, however, the tether forces dramatically increased with swelling and decreased with shrinking (Dai et al., 1998), supporting the tension hypothesis for surface area regulation.

In the Dai and Sheetz (1995) study, they used coated spheres to probe the growth cone membrane in order to determine the tether forces and membrane viscosity. The results also demonstrated that actin cytoskeleton affects the viscoelastic behavior of the membrane but also the force required for membrane extension. These results shed new light on our understanding and quantification of the neuronal membrane mechanical properties. Similar studies using this method followed, clarifying the tethering and growth processes taking place (Li et al., 2002; Ermilov et al., 2004; Nussenzveig, 2018; Hochmuth et al., 2020; Soares et al., 2020).

Further probing of neuronal cytoskeleton physical properties with OT includes the study of the dynamics and the measurements of forces exerted by lamellipodia and filopodia (Cojoc et al., 2007), cytoplasmic projections at the extreme edges of the growth cone (Figures 4a,b), which probe the rigidity and composition of the environment. Remarkably, Amin et al. (2011) have used OT to identify the elementary events of lamellipodia dynamics. Looking at the Brownian motion of optically trapped beads attached to lamellipodial membrane, they measured the distribution of the beads' velocities, and calculated the “jumping” times to be between 0.1 and 0.2 ms. They also measured the frequencies and amplitudes of those jumps and measured their changes in the presence of different molecules. Another interesting example is a study by Cojoc et al. (2007), where they placed a trapped bead against isolated filopodia and lamellipodia and measured single filopodial forces not exceeding 3 pN, and lamellipodial forces of at least 20 pN. These results proved that an isolated filopodium does not have the capacity to alter the environment, which explains why it changes its direction of growth when encountering large objects. Lamellipodia on the other hand, can apply substantial forces, and can move or lift large structures in order to grow further in a chosen direction. While filopodia simply explore their environment, lamellipodia can exert significant forces to mechanically modify the environment and facilitate the growth of axons.

It is worth noting that other methods to study neuronal growth involve the direct guidance of growth with OT by placing optical traps near a lamellipodium and optically pulling it (Ehrlicher et al., 2002; Mohanty et al., 2005; Carnegie et al., 2008; Graves et al., 2009). However, this approach may be confounded by the effects of heating by the laser beams used for OT; it is disputed whether the neuron's guidance is due to thermal effects rather than an optical force gradient (Stevenson et al., 2006; Ebbesen and Bruus, 2012). While OT may apply optical gradient forces on neuronal axons toward the trap focus, the heat generated within the trap focus may also trigger a biochemical signaling cascade due to the heating of the cell membrane, which results in a chemical guidance of cell growth (Henley and Poo, 2004).

A less direct method of neuronal guidance is the creation of localized microfluidic flow using OT (Wu et al., 2011). By changing the rotation direction and location of a trapped particle, Wu et al. (2011) have been able to create a local flow around the particle and consequently a shear force that influenced the growth cone's development, showing that the environmental dynamics are influencing neuronal growth.

While neuronal growth can be influenced and guided by physical or chemical factors, neurons have shown preferences in their connectivities. Clarke et al. (2008) have optically manipulated retinal neurons with OT, and have shown that cone and rod cells have different target preferences. Using OT, they isolated retinal cells and formed pairs of first order photoreceptor cells (rods and cones) with second or third order neurons. By analyzing the direction and amount of neuritic growth, they found significant differences in cone and rod cells' intrinsic preferences (Figure 4c), which could help explain the natural patterning of photoreceptors on the retinal layers.

### 3.4. Mechanical Properties of Neurons

Another essential research area in neuroscience is the investigation of morphological development of the brain and responses to injury. Recent studies suggest that the mechanical properties of the brain deeply influence neurodevelopment (Bayly et al., 2014; Budday et al., 2014), and are correlated with developmental disorders such as lissencephaly and polymicrogyria, where brain folding are abnormally reduced or increased (Raybaud and Widjaja, 2011), brachycephaly and plagiocephaly, where the brain has a flat or asymmetric shape (Hutchison et al., 2004).

**Figure 5:**
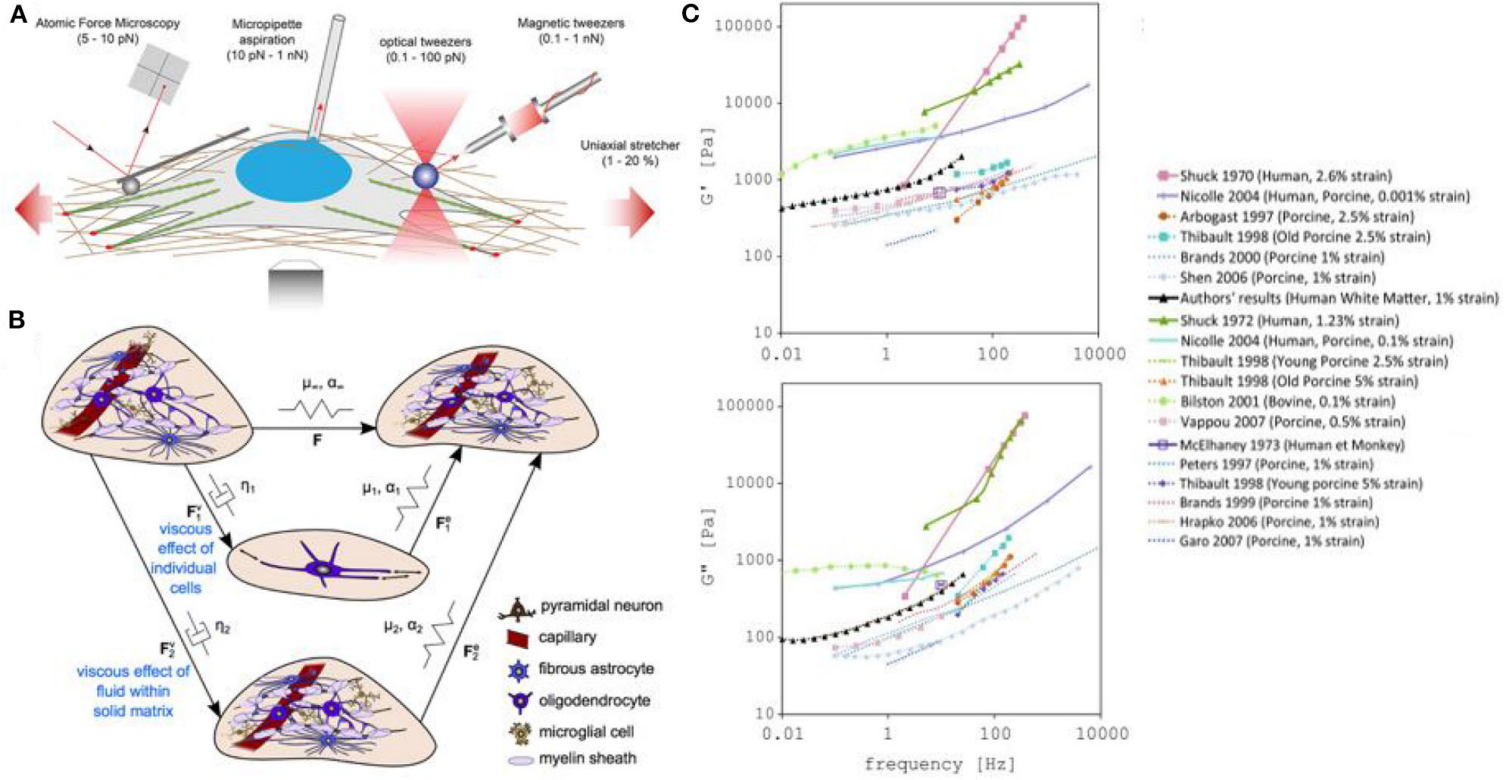
Measuring and modeling the mechanical properties of neurons. **(A)** Examples of force-application techniques used to probe the rheological properties of cells or to apply well-defined external loads, reproduced from Mohammed et al. (2019) (CC BY 4.0). **(B)** Model of elastic networks and viscous dampers in the brain. From Budday et al. (2020) (CC BY 4.0). **(C)** Storage (G′) and Loss modulus (G″) measurements in different models (including human, monkey, calf, porcine, bovine). From Forte et al. (2017) (CC BY 4.0).

To understand the mechanisms that drive neurodevelopment and cause neurological disorders, it is essential to understand the biomechanics, or rheological differences between healthy versus unhealthy brains, as well as differences within brain regions. While indentation methods have been used to physically deform brain slices in order to measure the physical response, stiffness, and elasticity of specific areas of the brain (Budday et al., 2015; Antonovaite et al., 2018), this method lacks precision and the capability to perform measurements *in vivo*. More precise and recent techniques such as atomic force microscopy, micropipettes, optical tweezers, magnetic tweezers, and uniaxial stretchers, have allowed great progress into mechanotransduction pathways studies (Huang et al., 2004; Chighizola et al., 2019) (Figure 5A). Amongst these methods, OT offers the advantage of being non-invasive, applying comparively large forces, being able to probe cells in a 3D scaffold environment and precisely measuring forces in 3D space (Nawaz et al., 2012; Capitanio and Pavone, 2013; Pontes et al., 2013; Arbore et al., 2019; Li et al., 2020). Very recently, Dagro et al. (2019) used optically trapped silica beads to deform cell surfaces and measure their stiffness and elasticity. They successfully measured the elastic properties, at both high and low strain rates, of glial cells, opening a new avenue for the precise measurement of the mechanical properties of brain tissue.

In the case of traumatic brain injuries, the leading cause of death and disability in children and young adults, there is an increasing need for a better understanding of the process of injury development in the brain, and the development of effective protective measures. In recent years, an effort to better understand traumatic brain injury dynamics has been undertaken, involving computational models of the head and brain. In particular, the measurement of the stiffness of the brain tissue has been thoroughly investigated by measuring the storage modulus (G′ or elastic portion of the modulus) and loss modulus (G″ or viscous portion of the modulus) of the brain using mechanical techniques such as compression or shear quasi-static methods (Chatelin et al., 2010; Budday et al., 2020). However, the lack of accurate datasets and differences in brain mechanical property measurements have complicated the development of realistic models (Figures 5B,C). While we can explain these value variations as being caused by tissue heterogeneity, brain anisotropy, species differences, age variations, or differences in experimental parameters, a new method for precisely measuring brain biomechanics remains of great interest.

Magnetic Resonance Elastography (MRE) has been popular because it is non-invasive, and allows measurements from a living organism. However, this method lacks spatial resolution, which results in poor measurement accuracy and high variance for small regions of the brain (Johnson et al., 2016). A solution for this spatial accuracy limitation is the use of FOTs, a method which also has limited experimental variation. In a recent study by Chiang et al. (2019), they have fabricated and optimized FOTs for brain tissue mechanical stiffness measurements and obtained three reliable data sets for white matter that agree with published results. This new method should be considered actively in this area.

### 3.5. Probing Sensory Structures and Whole-Brain Networks

On the larger scale, we know that the brain constantly senses stimuli, processes information, and makes predictions based on the physical environment. Numerous studies in this area have allowed great advances in the determination of the brain regions involved in sensory perception and processing. However, the full information processing network is currently a mystery and therefore of great interest.

In particular, efforts have been made into the study of mechanotransduction of neurons, which use specific organelles (hair cells for instance) to detect a wide range of mechanical forces and frequencies, and are the origin of crucial senses such as hearing, touch, proprioception, and noxious mechanical sensation. While indentation pipettes, pressure jets with a pipette, or microfluidics, are used to pull and push cells to provide local mechanical stimulation (McCarter et al., 1999; Sánchez et al., 2007; Desmaële et al., 2011; Thompson et al., 2016; Vanwalleghem et al., 2020), OT is, once again, highly desirable as it has the advantage of a very precise probing, provides simultaneous measurement of the applied force and deformation (Mohammed et al., 2019), and, as mentioned before, is totally contact free. In particular, past studies have used OT to stimulate mechanoreceptors (Li et al., 2002; Ermilov et al., 2004; Rodríguez, 2019), revealing their response characteristics, as well how their mechanical properties change in presence of chemicals or variable electrical potentials. As an example, Li et al. (2002) used optically trapped polystyrene beads tethered to the membranes of outer hair cells to measure their mechanical characteristics. They found the average force to and from a plasma membrane tether at the lateral wall of the hair cell to be large: 499 ± 152 pN; about 3.5 times greater than that at the basal end of the cell: 142 ± 49 pN. These results are consistent with the presence of a more extensive cytoskeleton supporting the plasma membrane at the site of the lateral wall.

**Figure 6:**
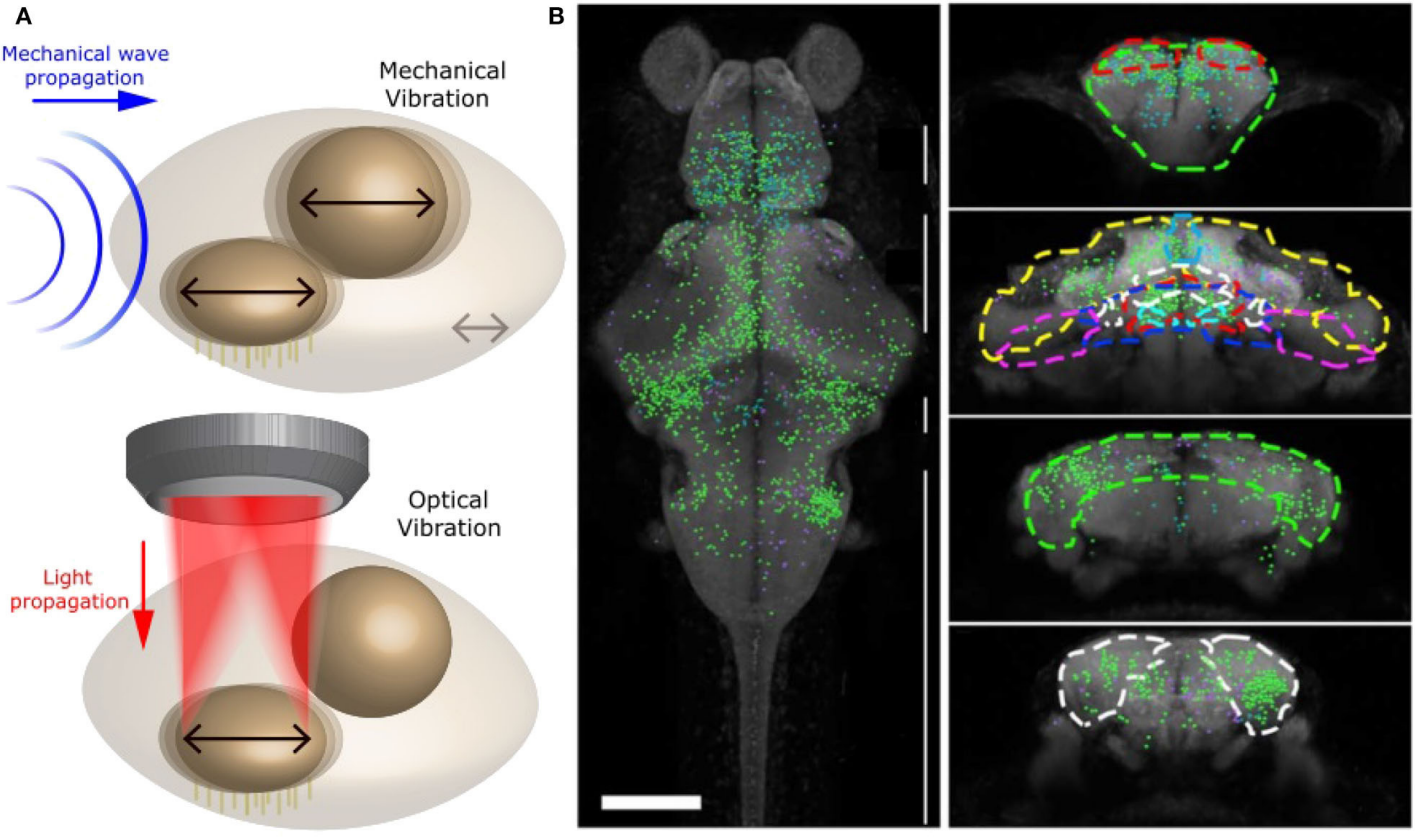
Optical manipulation of mechano-receptors. **(A)** Top: Schematic of a mechanical wave (blue) traveling through zebrafish inner ear and vibrating sensory organs (black arrows). Bottom: Schematic of OT manipulating selectively one sensory organ within the inner ear and producing a similar vibration to a mechanical wave. Reproduced from Favre-Bulle et al. (2020) (CC BY-NC-ND 4.0). **(B)** Distribution of neurons involved in vestibular processing in larvae zebrafish brain. Left: Top view of whole brain. Right: coronal view of specific sections of the brain, adapted from Favre-Bulle et al. (2018), Copyright 2018, with permission from Elsevier.

These studies, however, were done *in vitro* and did not interrogate the full brain and network. In our recent studies (Favre-Bulle et al., 2017, 2018, 2020; Taylor et al., 2018), we have successfully applied forces to zebrafish otoliths: ear-stones located in the inner ear. In particular, we have been able to apply OT to each of the four otoliths of 6 days old zebrafish embryo (Figure 6A), and study the behavior (tail bends and eyes rolls) and brain activity in response to individual or multiple otoliths optical manipulation in experiments up to 30 min in length. This was performed by combining OT with bright field imaging and Selective Planar Illumination Microscopy (SPIM). Since the utricular otolith is known to be the main actor in the detection of acceleration, we have applied OT to the utricle otolith with different directions and magnitudes. Interestingly, we have shown that the fish was compensating behaviorally for the perceived, but non existing, body acceleration. By activating only one ear with OT, a manipulation that is not possible with natural sound, we have also shown that the neuronal network of individual ears project to the contralateral ear, as previously shown in different models, and that responsive neurons showed responses profile dependent on OT configuration across whole the brain (Figure 6B). The saccular otolith, on the other hand, is known to be deeply involved in the detection of sound. We have modified our OT system to allow higher frequency manipulations (10 Hz to 1 kHz) to produce Bio-Opto-Acoustic (BOA) stimuli (Figure 6A). Using the BOA technique, we have shown that we can displace all of the four ear-stones at a chosen frequency, that stimulate the neurons responding to natural tones. We have also revealed the integration and cooperation of the utricular and saccular otoliths, which were previously described as having separate biological functions, during hearing.

In other words, by combining OT with Selective Planar Illumination Microscopy (SPIM), we have been able to simulate acceleration and sound at variable frequencies, and therefore replicate natural vestibular and auditory stimuli (Figure 6A and Favre-Bulle et al., 2018, 2020). Since OTs offer high spatial precision, we could manipulate single elements of the inner ear and precisely map the neural networks that responded, providing important information about the separate and shared circuits involved in hearing and vestibular perception.

## 4. How Far Could OT Go in Neuroscience?

OT, although only recently applied in neuroscience, has already provided valuable insights into the functioning and behavior of neurons on scales from whole organisms down to single molecules. Despite these successes, there are still several limitations preventing the broader use of OT in neuroscience. Current limitations are largely related to their use *in vivo*, which requires controlled and precise OT deep in the turbid and dynamic medium of the brain. In this section, we describe recent advances in optics and computation that could be useful for designing the next generation of OT neuroscience experiments.

### 4.1. Fiber Optical Tweezers (FOT)

Although FOT is not new (Constable et al., 1993), recent advances could make FOT a promising candidate for optical trapping deep within the brain. Much like a regular endoscope, FOT can be used to perform *in vivo* measurements while being minimally invasive. Unlike regular OT, FOT could be used to trap particles deep within the brain or for precise trapping near regions that could be damaged by regular OT. Most FOTs have a low numerical aperture, making it difficult to three-dimensionally trap a particle with a single FOT. In the future, this limitation may be circumvented using different types of fibers such as hollow core (Garbos et al., 2011; Bykov et al., 2015; Peng et al., 2020), or graded index fibers (Gong et al., 2013), and multimode fibers (Čižmár and Dholakia, 2011), as well as fibers with lenses (Li et al., 2016), plasmonic structures (Rodrigues Ribeiro et al., 2017), or photonic lanterns (Velázquez-Benítez et al., 2018) in order to extend the possible trapping configurations.

Recent advances in imaging through multimode fibers (Vasquez-Lopez et al., 2018) and computational tools that allow real-time beam shaping in multimode fibers (Plöschner et al., 2014) could be used to trap particles or structures deep within the brain (Čižmár and Dholakia, 2011). One of the main hurdles to overcome with imaging and trapping using multimode fibers is the sensitivity of the fiber to variations in temperature, pressure, or deformation, which can adversely affect the trapping or imaging quality. One alternative would be to use a rigid structure, such as a cannula (Kim et al., 2017), to reduce the sensitivity to environmental conditions, although this approach would be more intrusive and reduce the flexibility of the technique. Another alternative would be to continuously calculate the fiber's transfer matrix, which describes light transmission through the fiber. This could be done by using an approach similar to a *guide star* in adaptive optics: by placing a nano-particle or another suitable structure at the tip of the optical fiber, the experimenter gains a reference for calibrating the structure of the output light (Gu et al., 2015).

### 4.2. Computational Modeling

Computational modeling is an important tool for designing optical traps, understanding optical forces and torques, and modeling the dynamics of objects. Advances in computational power, availability of efficient and easy to use computer codes, and advances in algorithms for optimization and numerical modeling have all been beneficial to OT development.

Open source repositories, such as GitHub, make it easy to share and collaborate on computer codes for controlling and simulating SLMs (Bowman et al., 2017; Lambert, 2017; Aakhte, 2018; Lenton, 2020b), simulating optical tweezers (Herranen et al., 2020; Lenton, 2020a,c), and calculating light scattering (Roundy, 2020; Yurkin, 2020). For example, OTSLM (Lenton, 2020b; Lenton I. C. D. et al., 2020b) is a collection of simple patterns, iterative algorithms, and simulation methods for designing and modeling SLM patterns with a focus on OT. While many of the phase or amplitude patterns used to create structured light fields with SLMs in OT can be implemented with simple parametric functions (see Figure 3) or using iterative algorithms consisting of only a few lines of code (e.g., the Gerchberg–Saxton algorithm), it can still be very time consuming to search the literature for, and implement, these different patterns. Further still, implementation of more complex algorithms can be very difficult and time consuming, even when code is provided as supplementary material to the research papers describing the algorithm. The goal of OTSLM is to provide a free and open source repository for algorithms and patterns used in OT with examples, supporting documentation, a somewhat consistent interface, and freely available source code that anyone can use and contribute to.

While a major part of designing optical fields for OT is concerned with light shaping, another important part is calculating how a particle will orient itself in the optical field. This typically involves calculating the optical forces/torques that act on the particle and finding the equilibrium position/orientation. The most popular methods for calculating optical forces/torques in conventional OT are the T-matrix method (Nieminen et al., 2007; Herranen et al., 2017; Lenton, 2020a), geometric optics (Callegari et al., 2015), and for small weakly scattering particles, the dipole approximation or other zero-scattering approximations (Phillips et al., 2014). Recent advances have focused on combining these tools with dynamics simulations (Herranen et al., 2017; Lenton et al., 2018) and methods for calculating the non-optical forces such as the fluid dynamics or deformation of particles (Dao et al., 2003; Tapp et al., 2014). Simulating particles near walls, at the tip of optical fibers (i.e., FOT) or near plasmonic structures is often more complicated, and tools such as finite difference time domain (Yee, 1966; Benito et al., 2008; Lenton et al., 2017), discrete dipole approximation (Oskooi et al., 2010; Loke et al., 2011; Yurkin and Hoekstra, 2011), surface integral methods (Ji et al., 2014), or commercial packages such as COMSOL (Zhang et al., 2016; 2020a) and Lumerical (David et al., 2018; 2020b) are often used. Recent developments in machine learning have led to faster methods of simulating particles in OT (Lenton I. C. D. et al., 2020c), and faster hybrid algorithms for optimizing and simulating light scattering (Jiang et al., 2020). These advances could be useful in designing optical potentials that optimize certain OT properties such as trap stiffness and particle orientation.

### 4.3. Wavefront Shaping

While computational tools have enabled more intricate beams and OT beam shapes, the problem remains of how to project these traps deep within biological tissue. One of the main limitations of conventional OT for trapping within biological material is the nature of the biological matter itself. While organelles and membranes define the structure and function of cellular tissue, their irregularities and heterogeneities cause light distortions, rapidly degrading the light used for trapping or imaging. This makes it difficult to image or trap using conventional OT when samples are more than a couple of micrometers thick. For trapping of live cells and trapping *in vivo*, the problem is made more complicated as the tissue continuously evolves and changes. These are major problems both for imaging and optical trapping, and correspondingly there are numerous studies that explore different solutions (Park et al., 2018). Most of the methods used to deal with these problems were originally developed for imaging and then later applied to OT. Techniques for imaging in low-order scattering environments, such as *C. elegans* and zebrafish embryos, include optical coherent tomography (Huang et al., 1991), two-photon (Denk et al., 1990) and three-photon (Schrader et al., 1997; Rowlands et al., 2016) microscopy, and adaptive optics (Booth Martin, 2007). In high-order scattering environments, such as mice, wavefront shaping (Vellekoop and Mosk, 2007) and *guided star* based methods (Horstmeyer et al., 2015), speckle correlation (Bertolotti et al., 2012; Katz et al., 2014), scattering matrix measurement (Popoff et al., 2010; Choi et al., 2011), and various holographic techniques (Papadopoulos et al., 2016) are used to precisely reconstruct an image. The problem of generating a high-resolution image is very similar to creating a tightly focused optical trap; as such, these methods have been applied to OT (Dholakia and Čižmár, 2011; Zhong et al., 2017) and later to neuroscience (Shoham, 2010; Yoon et al., 2020).

A recent method, called focus scanning holographic aberration probing (F-sharp), has given promising results for neuroscience. This method is minimally invasive. It is based on holography and involves measuring the phase and amplitude of the scattered electric-field point spread function in order to determine the wavefront correction. Recent results (Papadopoulos et al., 2020) show imaging of neuron bodies, and partial axons, located 400 μm under a thinned skull of 5-week-old mice. While this method has thus far been used to improve imaging in mice, it should also improve capabilities in imaging and optical manipulation in adult zebrafish and other animals with similar sized brains.

Another difficult problem in wavefront shaping is optimizing patterns that selectively illuminate certain areas while not illuminating others. When trapping biological material or combining OT with other imaging techniques, it may be necessary to avoid illuminating certain regions of a sample to avoid, for example, heating, photodamage, or photobleaching. For conventional OT, one solution is to create beams using an SLM and an appropriate iterative algorithm that optimizes some function describing both regions where light should be and regions that should remain dark. As mentioned earlier, advances in faster simulations could be useful for optimizing with respect to the optical trap properties in addition to optimizing for the shape of the light field. These sorts of optimizations can be limited by the models used for describing the environment surrounding the particle. Another alternative is to use more localized optical fields, such as plasmonic tweezers or FOT.

### 4.4. Force Measurement

As discussed in previous sections, OT is a valuable tool for measuring the forces exerted by various cells and membranes, for example Cojoc et al. (2007) and Dai and Sheetz (1995). Force measurements with OT are useful for determining other mechanical properties such as the mechanical stiffness or viscoelasticity of various cells and their surrounding environments. There are numerous methods for measuring the force, most involving assumptions about the trap shape (such as assuming a linear restoring force), calibrating using a known force, or collecting a significant portion of the scattered light in order to directly estimate the force from the scattering distribution (Jun et al., 2014; Thalhammer et al., 2015; Bui et al., 2018). When trapping and measuring particles deep inside samples, it is not always possible to use the same force measurement techniques: thicker samples can lead to larger aberrations affecting the trap shape, it may not be possible to generate a known force for calibration, and a significant portion of the light may be absorbed or not be measurable. The main solutions to these problems are related to the advances in FOT, computational modeling and wavefront shaping that have been previously discussed. For example, the effect of aberrations (which can negatively affect how both the shape of the optical potential and measurements of the scattering distribution) could be reduced by reducing the distance between the particle and the lens using FOT or using adaptive optics to compensate for the distortion.

Another cognate advancement that has important implications for force measurements is the recent advance in algorithms and the application of machine learning to particle tracking and recognition (Helgadottir et al., 2019; Fränzl and Cichos, 2020; Rose et al., 2020). Packages such as DeepTrack (Helgadottir et al., 2019, 2020) allow for fast accurate tracking and classification of particle mixtures. Force measurement techniques that involve measuring the position of a particle in the optical trap can greatly benefit from these improved algorithms for particle tracking. Although position based force estimation often assumes a linear potential, it is also possible to apply these same techniques when position and force do not have a linear relationship. This usually involves using the thermal motion of the particle to calibrate the optical potential. Methods such as FORMA (García et al., 2018; García, 2019) enable estimation of both the conservative and non-conservative parts of the optical potential. When the scenario can be accurately modeled, it is sometimes possible to fit the available experimental measurements to the model in order to estimate the optical force. These kinds of computational models require precise information about the particle properties and the properties of the surrounding material as well as enough data and fast enough simulations to be able to fit the experimental measurements to the model. Recent studies have looked at different methods for fitting models to experimental data both in the damped and underdamped regime (Brückner et al., 2020; Frishman and Ronceray, 2020). The amount of information that can be extracted from the Brownian motion of a particle was recently considered by Frishman and Ronceray (2020).

For photonic force microscopy, one of the main limitations is related to the properties of the optical probe and how easily it can be trapped and orientated. The resolution of photonic force microscopy is largely related to the size and shape of the probe: large probes have low resolution due to the large contact area with the sample but are less affected by thermal motion and are easier to trap/detect; while small probes can achieve higher resolution due to the smaller contact area but at a cost of a lower signal to noise ratio from weaker trapping and larger effects of thermal noise. One solution is to use large non-spherical probe particles with very finite tips (similar to the tips used in atomic force microscopy). Recently, Desgarceaux et al. (2020) demonstrated that large numbers (~ 10^7^ probes per batch) of similar probes which a diameter of almost 2 μm and a pointed tip of 35nm can be fabricated and stably trapped, allowing high resolution and low signal to noise measurement of the surface structure of a infected red blood cell. This same approach could be applied to scanning force measurements of cells and membranes in a neurological context.

### 4.5. Molecular Studies With OT

Most current studies of molecules involved in brain function involve using OT *in vitro*, either using probe particles attached to molecules (Capitanio and Pavone, 2013), or plasmonic devices for label-free OT (Huang and Yang, 2015; Choudhary et al., 2019). For label based approaches, most advances will likely come from advances in sensing and functionalization of biomolecules, extending the range of molecules that can be trapped and sensed. Trapping and sensing inside cells, or deep within the brain, can be difficult for label based methods since it requires inserting a suitable probe/label into the environment. Hollow core FOT could be useful for delivering plasmonic particles for use as probes (Garbos et al., 2011), the FOT could then be used for subsequent trapping/sensing. Another solution is to use label-free trapping and sensing using plasmonic OT in combination with FOT (Ehtaiba and Gordon, 2018, 2019), in order to create traps/sensors that could potentially be inserted deep into a sample or integrated into a *lab on a chip* platform. Another recent development is optical tweezers-in-tweezers, consisting of a plasmonic OT held in place by a regular OT (Ghosh and Ghosh, 2019; Wills, 2019). This method could be useful for the precise delivery of molecules to neurons, and be an alternative to the method demonstrated by Pinato et al. (2011) who used micro bubbles to deliver molecules to neurons.

## 5. Outlook

Most of the methods and techniques discussed in this review have been implemented *in vitro* for the study of small scale effects. However, with currently emerging technologies, we can foresee these studies moving *in vivo* with visualization of localized stimulation effects on the large scale. Currently, *Drosophila, C. elegans, D. translucida*, zebrafish, as well as hydra and brain organoids (if we consider loose neuron systems), are models where existing technologies have allowed whole brain imaging with cellular resolution. Therefore, we can imagine studies where the full neuronal network of senses is recorded while delivering localized stimulation. An example of possible studies is the determination of mechanotransduction systems networks, such as touch. *C. elegans* have six touch receptor neurons scattered around its body. We can imagine the manipulation with OT of these touch receptor neurons, individually or in concert, and the simultaneous imaging of the full neuronal network. Similarly, we can imagine the manipulation with OT of hair cells around a zebrafish embryo. These possible studies would allow great advances in the determination of the network for touch and flow sensing.

Another interesting area is the investigation of the brain-gut axis. Very recently, Kaelberer et al. (2018) have found enteroendocrine cells in mice that project into the vagal nerve, thereby communicating with the central nervous system. While the enteric nervous system was originally believed to transmit information to the central nervous system via hormones, this study revealed a more direct circuit for gut-brain signaling. While the gut-brain relationship is an active area of research using more traditional approaches (Ezra-Nevo et al., 2020; Spencer and Hu, 2020), we can imagine combining OT with wavefront shaping to manipulate bacteria and nutrients in the digestive system or the nerve cells lining the gut, and imaging the brain activity simultaneously. These studies would allow the precise study of bacteria-nutrients-enteroendocrine interactions, as well as their repercussions on brain activity, brain states, and behavior.

OT has already been invaluable in neuroscience, enabling various studies on neuronal growth, function, and communication on a molecular, cellular, and whole organism scale. Further still, the future is bright for OT in neuroscience. As advances in optics, computation, and OT techniques gradually make their way into neuroscience, we can expect OT to become more prominent in the field, especially for *in vivo* studies in larger models. The combination of OT and adapted wavefront shaping will allow the achievement of OT deeper than ever in biological organisms and will likely lead to *in vivo* studies in adult zebrafish and mice. Advances in plasmonics are leading to label-free trapping and sensing of a greater range of molecules. Combined with FOT or tweezers-in-tweezers technologies, these tools could lead to applications of optical trapping and sensing deep within the brain, or very precise delivery of molecules and proteins directly to parts of neurons.

One of the major advantages of OT is how easily it can be combined with other techniques such as magnetic or acoustic trapping techniques for applying larger scale forces, or different imaging and microscopy techniques. Further advances in plasmonics, fibers, and wavefront shaping will be important for combining OT with other imaging or manipulation techniques, such as for selectively illuminating certain regions of a sample or to enable tweezers in locations where conventional OT systems simply wouldn't fit. OT continues to learn and borrow from other fields, incorporating advances in adaptive optics and light shaping techniques from microscopy and consequently achieving greater resolution, improved trapping, and greater accuracy. At the same time, with advances in force measurement techniques using computational models or with detectors that measure the momentum distribution of the scattered light, we expect OT measurements to become more precise and more flexible. As the flexibility of OT and range of measurements that can be performed with OT continues to advance, we can expect these technologies to continue to be adapted for studying different aspects of the brain and its function.

## Code Availability

Code used to generate Figure 3 can be downloaded from^1^.

## Author Contributions

IL and IF-B wrote the original manuscript. All authors contributed to drafting and editing. IL designed and ran simulations for Figure 3.

## Conflict of Interest

The authors declare that the research was conducted in the absence of any commercial or financial relationships that could be construed as a potential conflict of interest.

## Footnotes

^1^https://github.com/ilent2/ot-neuro-review-supp

## Supplementary Material

Supplementary Video 1 A particle (blue circle), initially outside a moving OT, falls into the OT and is dragged by the OT as it goes around in a circle. Particle displacement track is shown in white.

Supplementary Video 2 Distribution of positions (white dots and blue bars) of a particle (green dashed circle) in a counter-propagating OT using feedback in the horizontal direction: the three panels show (from left to right) a trap with a high stiffness, a trap with a low stiffness and a trap with two equilibria, all generated using the feedback system.

## Abbreviations

OT: optical tweezers
FOT: fiber optical tweezers
SLM: spatial light modulator
NCS-1: (protein) Neuronal calcium sensor 1
Sema3A: (protein) Semaphorin 3A
Netrin-1: (protein) Netrin 1.
